# Participatory science and innovation for improved sanitation and hygiene: process and outcome evaluation of project SHINE, a school-based intervention in Rural Tanzania

**DOI:** 10.1186/s12889-017-4100-7

**Published:** 2017-02-07

**Authors:** Erin Hetherington, Matthijs Eggers, Joyce Wamoyi, Jennifer Hatfield, Mange Manyama, Susan Kutz, Sheri Bastien

**Affiliations:** 1 Cumming School of Medicine, University of Calgary, TRW 3rd Floor, 3330 Hospital Dr. NW, Calgary, AB T2N 4N1 Canada; 2 Maastricht University, School for Public Health and Primary Care (Caphri), P.O. Box 616, 6200 MD Maastricht, The Netherlands; 3 National Institute for Medical Research, P.O Box 1462, Mwanza, Tanzania; 4 Weill Cornell Medical College in Qatar, Education City, Qatar Foundation, P.O Box 24144, Doha, Qatar; 5 Faculty of Veterinary Medicine, University of Calgary, Health Sciences Centre, 3330 Hospital Dr. NW, Calgary, AB T2N 4N1 Canada; 6 Department of Public Health Science, Faculty of Landscape and Society, Norwegian University of Life Sciences, Post Box 5003, Akershus, 1432 Ås Norway

## Abstract

**Background:**

Diarrheal disease is a major cause of mortality and morbidity in low and middle income countries with children being disproportionately affected. Project SHINE (Sanitation & Hygiene INnovation in Education) is a grassroots participatory science education and social entrepreneurship model to engage youth and the wider community in the development of sustainable strategies to improve sanitation and hygiene.

**Methods:**

Based in rural and remote Tanzania, this pilot study engaged pastoralist high-school students and communities in the development and evaluation of culturally and contextually relevant strategies to improve sanitation and hygiene. Using a train-the-trainer approach, key activities included teacher workshops, school-based lessons, extra-curricular activities, community events and a One Health sanitation science fair which showcased projects related to water, sanitation and hygiene in relation to human and animal health. The process and outcome of the study were evaluated through qualitative interviews and focus group discussions with diverse project participants, as well as pre- and post- questionnaires completed by students on knowledge, attitudes and practices concerning sanitation and hygiene.

**Results:**

The questionnaire results at baseline and follow-up showed statistically significant improvements on key measures including a decrease in unhygienic behaviors, an increase in the perceived importance of handwashing and intention to use the toilet, and increased communication in the social network about the importance of clean water and improved sanitation and hygiene practices, however there were no significant changes in sanitation related knowledge. Qualitative data highlighted strong leadership emerging from youth and enthusiasm from teachers and students concerning the overall approach in the project, including the use of participatory methods. There was a high degree of community engagement with hundreds of community members participating in school-based events. Sanitation science fair projects addressed a range of pastoralist questions and concerns regarding the relationship between water, sanitation and hygiene. Several projects, such as making soap from local materials, demonstrate potential as a sustainable strategy to improve health and livelihoods in the long-term.

**Conclusions:**

The Project SHINE model shows promise as an innovative capacity building approach and as an engagement and empowerment strategy for youth and communities to develop locally sustainable strategies to improve sanitation and hygiene.

## Background

Diarrhoeal disease is a major cause of mortality and morbidity in low and middle income countries with children being disproportionately affected [1]. Chronic diarrhoea can result in malnutrition which may have negative consequences for child growth (stunting and under weight) and cognitive development, with subsequent impacts on human capital and productivity which span the life course [2–5]. Open defecation, which is still practiced by approximately one billion people, poor hygiene practices, and lack of access to safe drinking water and adequate sanitation contribute substantially to the burden of diarrhoeal disease in low- and middle- income settings [6, 7]. Rural areas are particularly at risk due to limited infrastructure and the difficulty of carrying out interventions to improve sanitation and hygiene practices in remote areas.

The Millennium Development Goals (MDGs) aimed to reduce the proportion of people without access to safe drinking water and basic sanitation facilities by half. However, despite progress in this area, global estimates indicate that 2.4 billion people are still using unimproved sanitation facilities including almost 1 billion who practice open defecation [8, 9]. The importance of water and sanitation to development outcomes received greater attention and focus in the new Sustainable Development Goals, in particular Goal 6 “Clean Water and Sanitation”, which underscores the need to end open defecation and increase community participation in improving sanitation and hygiene management [10].

Whilst providing improved infrastructure is a key element of any effort to improve sanitation and hygiene, individuals and communities may opt not to make use of facilities for a number of reasons [11–13]. Practices related to sanitation and hygiene may be influenced by knowledge and attitudes, among a host of other socio-cultural, economic, community and structural level factors.

Schools are an important and cost-effective setting for water, sanitation and hygiene (WASH) interventions, particularly in light of increases in school attendance worldwide as a result of Universal Primary Education initiatives which provide free access to primary schools. Large numbers of young people, but also parents, teachers and the wider community can be reached with school-based interventions. Reviews of studies of the impact of school-based WASH interventions show mixed results on outcome measures such as increased knowledge, attitudes and practices, decreased absenteeism and infection and point to a need for rigorous research on both the impact of interventions, as well as details on the implementation process [12, 14, 15].

Evidence shows that health promotion interventions are more likely to be effective if they are grounded in social and behavioural science theory, to help link the pathway to a desired outcome [16]. There are a small but growing number of studies in the peer reviewed literature regarding theory-based WASH interventions [15, 17]. The aim of this paper is to present the results from both the process and outcome evaluation of an intervention targeting pastoralists in rural Tanzania. Pastoralists rely on raising animals for their livelihood, are often semi-nomadic and typically reside in dry arid areas that experience drought and lack of access to other resources and services. To the best of our knowledge, this is the first study in the peer-reviewed literature that investigates pastoralist sanitation and hygiene-related knowledge, attitudes and practices. We anticipate that the evaluation of this intervention may assist future program planners to improve future school-based sanitation and hygiene interventions and spur further studies of this issue.

### Project overview

#### Project setting

The study was conducted in two secondary schools in the Ngorongoro Conservation Area (NCA), Tanzania. The NCA is a UNESCO World Heritage Site, which holds the unique position of serving as a tourist destination for wildlife enthusiasts, as well as traditional homeland for Maasai pastoralists who are semi-nomadic and live in close proximity to their livestock.

Project SHINE (Sanitation and Hygiene INnovation in Education) is a participatory action research project that was developed as part of a long-term transdisciplinary research collaboration between the University of Calgary, Canada, the Catholic University of Health and Allied Sciences, Tanzania and communities of Maasai pastoralists in the NCA, Tanzania. The team’s research is situated within a One Health paradigm which focuses on the interrelationship between humans, livestock and the environment [18]. The development of Project SHINE stems from community concerns regarding the impact of parasitic infection on child health and records from a rural hospital in the area which indicate that helminth infections and protozoa are a major public health concern.

#### Overview of the intervention

The development and theoretical framework for this pilot study has been described in detail elsewhere [19]. Briefly, Project SHINE is situated in a social ecological framework and draws on the Integrated Behavioural Model for Water Sanitation and Hygiene framework (IBM-WASH) [12, 20]. The IBM-WASH framework uses a multi-level approach which takes into account relevant contextual, psychosocial and technological factors. Project SHINE purposefully uses a positive participatory approach which honours traditional knowledge and promotes non-stigmatizing activities to empower youth and communities to develop strategies to improve sanitation and hygiene [19, 21]. This is in contrast to other models, some of which incorporate shaming and disgust techniques to promote behaviour change [22]. The project was designed to fit into the existing science and civics curriculum to minimise teacher burden.

The intervention aimed to achieve the following objectives: 1) improve knowledge, attitudes and practices among students related to sanitation and hygiene, as well as increase interest and motivation for science and 2) engage secondary school students and the wider community in the development and evaluation of sanitation and hygiene prototypes and health promotion strategies.

**Fig. 1:**
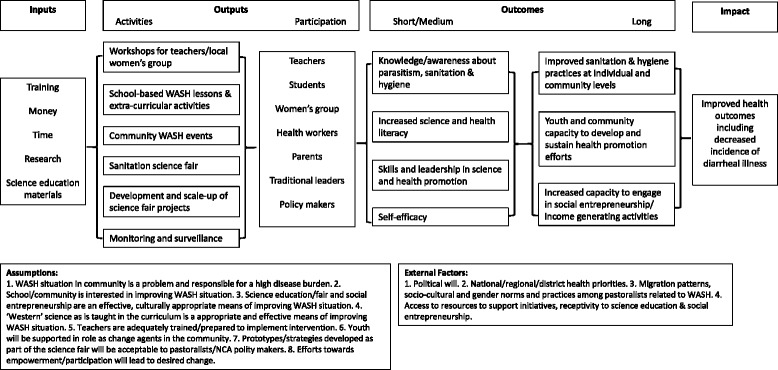
Project SHINE logic model

This pilot study took place in the two secondary boarding schools in the NCA between May and November, 2014. All registered students were invited to participate in the baseline and follow-up study, and the intervention was intended to target primarily Form 3 students (Grade 9). The headmaster at each school assigned a teacher to be the Project SHINE coordinator whose main tasks included receiving and communicating information about the intervention. The full logic model for Project SHINE can be seen in Fig. 1. Essentially, Project SHINE applied a “train the trainer” model designed to engage students and teachers in WASH activities, and for the schools to act as a hub of knowledge and innovation to engage the wider community. Teachers participated in workshops with initial training in participatory techniques for engaging students on issues related to improving sanitation and hygiene. There was a focus on developing low-cost, low-tech strategies that would be culturally and contextually relevant (also known as “frugal innovation”). For instance, frugal tools such as the ‘Foldscope’, an origami-based microscope that costs less than a dollar to manufacture, were distributed to teachers and students. The aim of introducing such an innovation was to equip and empower participants to perform scientific sanitation and hygiene-related investigations, but also to inspire them to think creatively and innovatively about how they could solve problems using limited resources in their own context. Teachers delivered lessons to students, and highly engaged students formed “SHINE clubs”, with representatives appointed by teachers from each Form, who were tasked with communicating the lessons to school and community members and hosting school wide community events. The SHINE club members were given t-shirts with the SHINE logo, as well as small stipends for each club (60,000Tsh, equivalent to $30USD) to purchase materials for their outreach sessions. All Form 3 students developed a project for a sanitation science fair which was an opportunity for students to pose their own research questions. Since many students come from pastoralist families, they were encouraged to use a One Health approach, which considers the impact of water, sanitation and hygiene on both human and animal health [23]. For instance, projects included investigation into the presence of parasites in cow dung, biogas experiments and social science related projects looking at the management of water sources between livestock, wildlife and humans. The sanitation science fair served as an important platform to showcase the projects and innovative ideas to the rest of the school and the wider community, which were designed to improve sanitation and hygiene in their community.

## Methods

### Community consultation

The preliminary framework and strategies used in the project were developed in collaboration with community members as a result of the research team’s long standing relationship with the community. As a more formal method of community consultation, a series of ‘Think Tanks’ were carried out at the onset of the project and twice during implementation. Research team members met with key stakeholders including parents, hospital staff, village elders, government officials, and women’s group representatives to identify potential unintended consequences of the intervention. Community consultation also took place through the establishment of evaluation teams for the SHINE sanitation science fair which consisted of a broad cross-section of community members who were tasked with assessing student projects with particular emphasis on feasibility, relevance to the community and potential for social entrepreneurship and scale-up. Extensive field notes were taken during meetings before and after the science fair in order to understand community perspectives on the project and the science fair in particular.

#### Baseline and follow-up surveys

A theory-based quantitative survey measuring knowledge, attitudes and practices regarding sanitation and hygiene was administered at baseline (April, 2014) and immediately after the intervention (November, 2014) [19]. The development of the survey is described in detail elsewhere [19]. In brief, the survey was developed by the research team using a combination of previously developed scales and tools appropriate to this setting. Composite scales with sample questions, and reliability (Cronbach’s alpha) are described in Table 1 [24]. In addition to demographic information, the questionnaire included questions on knowledge, attitudes and practices about sanitation and hygiene. In addition, measures were included to tap into science interest and motivation, as well as engagement in health promotion planning [25, 26]. The majority of questions used a five point Likert scale response type. This population is linguistically complex, with the majority of students having *Kimaasai* as their mother tongue. Since *Kimaasai* is an oral language, the survey was provided in both *Kiswahili* and English, which are the languages of instruction in primary and secondary schools in Tanzania. The questionnaire was pilot tested at a secondary school in another district in the Arusha region with a similar demographic profile and revised accordingly. On the advice of community partners, because of the perceived sensitivity of some of the topics and to reduce the impression that the survey was a test, students answered the survey anonymously. The survey was administered in schools by the teachers involved in the SHINE project, with oversight by the principal investigator. Data were entered by research assistants from Tanzania and Canada using a standardised codebook, and 10% of the entries were double-checked for accuracy.

**Table 1:** Description of survey scales and sample questions

Scale	Number of items	Sample questions (responses on 5 point Likert Scales)	Cronbach’s alpha
Knowledge, Attitudes and Practices			
Knowledge	7	I believe that washing hands with water ONLY after using the bathroom is enough to protect from disease	0.426
		I think that if someone does NOT wash their hands with water and soap before eating it can lead to serious diseases	
Perceived Severity	1	If I got a worm infection, I would find this very serious	n/a
Perceived importance of washing	7	How important is it to wash hands with water and soap/mud/ash before eating	0.893
		How important is it to treat water before washing/bathing	
Frequency of washing	8	How frequently do you wash your hands with water and soap/mud/ash before eating	0.847
		How frequently do you wash your hands with water and soap/mud/ash after using the toilet/latrine	
Frequency of unhygienic behaviour	7	In the past 3 months, how often did you eat food without washing your hands first	0.798
		In the past 3 months, how often did you defecate outside and not use a latrine when one was available	
Self-Efficacy			
Self-efficacy to wash	2	How difficult is it to wash using soap/mud/ash after using the toilet/latrine	0.715
Intention to wash	2	How likely is it that you will use soap/mud/ash next time you use the latrine	0.736
intention to use toilet	1	How likely is it that you will use a toilet/latrine next time you need one, if one is available	n/a
Science interest and motivation			
Pros towards science	9	The science I learn is relevant to my life	0.867
		In science, I think it is important to learn to solve problems	
Cons towards science	3	I think science is boring	0.678
		I think that the science I learn about in school is not helpful in everyday life	
Communication			
Communication: hygiene	5	How often do you discuss washing hands after urinating and defecating with family and friends	0.776
		How often do you discuss washing fruits and vegetables with family and friends	
Student engagement in the classroom	4	In my class, teachers encourage students to participate in health education	0.752
		In my class, students are encouraged to ask questions in health education classes	
Student engagement in the community	2	In my community, young people are involved in planning local health education and promotion programs	0.704

Data were analysed using SPSS v. 22. Descriptive statistics including frequencies, means and standard deviations were generated. Highly skewed variables were transformed. For each construct (e.g. knowledge of hygiene practices) mean scores were calculated. Changes from baseline to follow-up were analyzed using independent t-tests to compare mean scores at baseline and follow-up. One set of analyses examined the mean scores of the entire school, and another analyzed Form 3 students independently to see if there were larger changes among Form 3 students (the target grade for WASH lessons and the science fair). Differences between schools, sexes and age groups were investigated using Chi-square and t-tests. Given that students answered the survey anonymously, it was not possible to assess changes from baseline to follow-up using a within subjects design where measurement waves are matched per individual (dependent sample *t*-test). Instead, a between subjects design was used where each measurement wave was treated independently (independent sample *t*-test).

#### Individual interviews and focus group discussions

In November 2014, an independent Tanzanian-resident consultant (JW) travelled to the NCA to conduct interviews and focus-group discussions with study participants. Thirteen in-depth interviews were conducted with teachers involved in Project SHINE and one ward education officer. Six teachers were interviewed face-to-face in private offices in November 2014, and seven interviews were conducted in December 2014 over the telephone, due to scheduling issues in November. Four sex disaggregated focus group discussions were conducted with student members of the Project SHINE clubs, using a semi-structured topic guide that addressed perceptions concerning different topics, activities and approaches that were covered in the programme.

Interviews and group discussions were conducted in *Kiswahili* and transcribed verbatim. The transcripts were subsequently translated into English. The software NVivo version 10 was used to explore, organise and manage the data. Using a grounded theory approach, initial open codes were inductively developed by one coder (JW) and shared and further developed with the principal investigator (SB) [27]. During the first stage of analysis, open codes were assigned to segments of the transcripts to portray meaning. After all coding had been completed, axial coding was used to group the open codes into more abstract conceptual categories. The final coding structure was discussed and agreed upon by both the consultant and the principal investigator, and the consultant was responsible for the final coding of all transcripts.

### School Lessons

As part of the training workshops described above, teachers were provided with manuals which contained lesson plans and materials needed to conduct specific WASH lessons [19]. They were also given a teacher log book to fill out to capture information on which lessons had been implemented, the time needed for each lesson, and the successes and challenges associated with the lessons. Log books were collected and information from them was extracted and categorised into common themes and recommendations.

### Ethics

Ethical approval for this study was obtained from the Tanzanian national ethics board, the National Institute of Medical Research, and the University of Calgary Conjoint Health Research Ethics Board. The study used a tiered consent process whereby formal and informal leaders in the community were consulted and provided support for the study [19]. For students, a passive parental consent procedure was used whereby parents were provided with an information letter two weeks prior to the start of the study, which explained the purpose and nature of the study and which also provided contact information for the research team should they wish to pose questions. Passive parental consent procedures have been used for other school-based studies in Tanzania [28, 29]. The study team did not register any refusals to participate. For the interviews and group discussions, written consent to participate and to audio-record the sessions was obtained prior to the discussions.

## Results

### Process evaluation

A description of the activities undertaken, target audience reached and source of evidence are described in Table 2.

**Table 2:** Activities and sources of evidence

Activity	Description	Date	Delivered by	Target Audience	Evidence of implementation	Source of data
Teacher training workshops	Participatory workshops to share knowledge and enhance teacher capacity in: 1) parasitism; 2) sanitation and hygiene in local context; 3) basic water quality testing and treatment; 4) advanced water testing and organizing a science fair	May & Sept. 2014	SHINE core team^a^	Biology and civics teachers at both schools	20 teachers trained	individual interviews, participatory evaluation techniques at workshops
SHINE School lessons	Series of lessons on sanitation, hygiene, water testing and treatment adapted from workshops and teacher manuals provided to teachers	May to Oct. 2014	Biology & civics teachers	Form 3 students	At least 40 individual lessons delivered	log books, individual interviews, focus group discussions
Extra-curricular SHINE activities	Classroom lessons on sanitation, hygiene, water testing and treatment replicated by students to community members. School debates, additional cleaning schedule for toilets, and school grounds.	May to Oct. 2014	SHINE club members	Community members	At least 4 separate visits to deliver lessons in villages Two debates performed in front of the entire school. Additional cleaning schedule of latrines by SHINE club members.	log books, individual interviews, focus group discussions
Community WASH events	Three school-wide events with community members to mark World Water Monitoring Day, Global Hand Washing Day and World Toilet Day	Sept & Oct, 2014	SHINE club members	All school students and community members	Participation of 500–1000 students and community members per event	individual interviews, field notes
Sanitation Science Fair	Students designed and implemented experiments to address water, sanitation and hygiene issues	Nov, 2014	Form 3 students	All school students and community members	Participation of between 500–1000 including members of the Pastoralist Council (local government), traditional leaders, women’s group members and out-of-school youth.	individual interviews, field notes
Scale up of Science Fair projects	Additional training in youth-based social entrepreneurship and soap-making	Jan-May 2015	SHINE core team, WET Centre Zambia & Rocky Mountain Soap Company	Teachers, students and women’s group members	Not implemented until after end of formal project	Not applicable

^a^SHINE core team consisted of Tanzanian and Canadian graduate and undergraduate students and local translators and facilitators

With the exception of the scale-up of science fair projects as social entrepreneurship initiatives, all activities were completed during the six-month project period. It was not possible to measure this important component given the intervention timeframe and the school year which ends in November. The social entrepreneurship initiatives are however ongoing, and in particular, soap making in the community is being pursued through additional grants and other supports.

Table 2 demonstrates the “train the trainer” approach, with the initial workshops being delivered by SHINE core team members, with lessons and activities being delivered by teachers and then students themselves in both the school and wider community. There was active engagement from teachers at both schools with 20 teachers completing the workshops and delivering at least 40 lessons to students. SHINE club members (20 students in each school) then replicated lessons to community members in at least four separate villages. SHINE clubs also demonstrated positive sanitation and hygiene practices at WASH events which included participation of the entire school and invited community members. A One Health sanitation science fair was organised at each of the schools as a culminating activity in the intervention, and Form 3 students developed and carried out projects in teams. Each school science fair was attended by between 400 and 900 students, community members and local leaders.

#### Perceptions of SHINE intervention activities

Feedback from the interviews and group discussions suggested that project participants had enjoyed the various Project SHINE activities, including the participatory learning techniques that were taught in the workshops. For teachers, the SHINE workshops offered an opportunity to enhance their knowledge and develop skills to engage students in new ways of learning through the use of participatory techniques.

*I was personally very happy with the project. It provided us with the necessary knowledge, especially to the students, teachers and the non-teaching staff and were also able to spread the knowledge to other community members…The project also helped us to link between theory and practical sessions…*(Teacher 2).

Classroom sessions were perceived as important, with both teachers and students reporting that they found the participatory approach to learning a useful method of delivering the Project SHINE sessions.

*The students were very active on the activities and on methods used because are student centered methods* (Log book entry).

*And another lesson that we understood well was that on water treatment, for example we learnt how to use filtration instead of using methods that, that are not easy to get easily, even to use filtration, you use a clean piece of cloth, first you filter the water then you boil until it boils* (SHINE girls club, school 1).

Participants described how the project has had ripple effects not only on students and teachers, but also the wider community:

*It really helped the students a lot who in turn were able to teach the community members. For example, we hosted a small event here at school and the community members came where they were taught about various things like the importance of having latrines, how to use latrines, advantages of washing hands, how to keep the environment clean and how to store water. Students used different methods to teach like via pictorial presentation and by singing in order to deliver the message* (Teacher 8).

There were a number of extra-curricular activities which took place as part of the intervention, which were perceived positively, with one teacher reporting that the activities “*helped students apply the knowledge they already learnt in class. For instance, we made posters outside classrooms which reminded them to wash hands before eating or after using the toilets”* (Teacher 10)*.*

Teachers reflected on how the science fair was a novel approach and was well received by students. Science fair teachers talked about the fair as enhancing the interest of students in science as it increased their creativity, their concentration and their confidence. “*It was very good for the students we were actually glad because it made students gain more interest with the science subjects than before”* (Teacher 6).

One of the biology teachers remarked that the science fair was an important and very relevant activity in line with the biology curriculum which requires that Form 3 students perform a practical as part of the national examination, while another expressed awareness that teaching science in a practical, hands-on fashion improves examination performance in comparison to those that are taught through lectures alone.

Some teachers commented that they found the SHINE activities to be highly relevant and in line with their existing duties, “*…it has helped reduce teacher load because most things taught in the project are part of the school curriculum”* (Teacher 7). One teacher expressed the belief that the project has *‘simplified work for the teachers in teaching the students’* (Teacher 1)*.* However other teachers felt that the additional classes and extra-curricular projects created more burden in terms of teaching and timetable.

*The school timetable has been stretched a little bit. While the school timetable required us to do something else, at the same time we needed to do project activities that caused a conflict with the school timetable. But since the school headmaster/teacher was communicating with them so things were not…were not that bad* (Teacher 10).

### Challenges with implementation

The process evaluation also highlighted key challenges that hindered implementation including the timing of the intervention activities, communication between schools and local coordination, inadequate supplies and allowances to support intervention activities. There were also concerns around contextual relevance of some intervention activities.

Many teachers mentioned that they were not able to implement all components of the program because of limited time. Teachers talked about having to meet other school curriculum demands such as national exams while trying to also coordinate events for Project SHINE.

There was considerable dissatisfaction about the remuneration provided for workshops and a feeling that teachers needed more incentives. However, some teachers expressed appreciation for the resources brought to the schools as part of the project including the Foldscope and reflected that, “*the project has helped us with different tools like pipettes, flasks and Bunsen burners which were recently used in exams”* (Teacher 7)*.* Others would have liked additional equipment to deliver the project. “*This project was very good and it had just little obstacles, first the allowance money was little compared to the efforts put in, there was a bit of communication barrier between the project facilitator and people involved”* (Teacher 4). Even SHINE club students expressed that additional incentives to motivate members could help to improve the project. For instance, the distance between schools and villages made travel to conduct outreach sessions difficult and funds for diesel for the school vehicle were requested. In addition, the SHINE clubs felt that the small stipends provided were insufficient to develop the volume of materials necessary to reach as wide an audience in the community as possible.

Teachers perceived that the local management of the project could have been stronger and they noted that follow-up visits by the local part-time project coordinator were limited. They did however acknowledge that another challenging aspect to communication was lack of infrastructure and mobile network connections to facilitate improved communication. As one teacher remarked *“We have some problems with communication. Here at our area, there is no connection, so even when the coordinator calls, he may find us unreachable…”* (Teacher 11).

The reciprocal role of pastoralist social and cultural norms in facilitating or hindering project impacts sparked nuanced and varied debates among participants. For instance, several teachers remarked that certain cultural norms, values and practices that might pose a challenge to uptake of strategies to improve sanitation and hygiene, for instance one commented that the sharing of toilets across sexes and generations can be a difficult issue for pastoralist communities.

*It is usually very difficult to convince an old man who has lived for more than 50 years on the importance of using or having a toilet. They do not understand at all, they find it against their beliefs about sharing the same toilet with their children* (Teacher 11).

On the other hand, despite cultural norms, some immediate behaviours did change. A teacher noted that children stopped walking around without shoes, and another teacher reported that *“after this project, one of the elders here build himself a toilet”* (Teacher 4). Several teachers reflected on how difficult it is to change norms and practices in the short-term, but most commented that social change is indeed taking place in pastoralist communities and that the ideas and knowledge that stem from the project will leave an impact over time. Many remarked that such changes take time, while one commented specifically that:

*Maasai are very difficult to change, but because this project has been involving their leaders, for example I am a leader in the Maasai society, and also most of the people who have participated are leaders, and the Maasai pay much respect to their leaders, this will help to change…*(Teacher 3).

#### Outcome evaluation

Eight-hundred and fifty six students completed the baseline survey, and 826 completed the follow-up survey. We were unable to track specific loss to follow-up because both surveys were filled out anonymously, however, there were no differences in demographic characteristics, with the exception that the students were slightly older at follow-up. Table 3 shows selected characteristics of the students in both schools. Students’ ages ranged from 10 to 23 years with a mean age of 15.8. Since there are no previously validated or agreed upon objective measures of socio-economic status (SES) among the Maasai, various questions were asked to attempt to capture SES, including herd size and an asset index. Unfortunately, none of these measures displayed a satisfactory association with any of the outcomes or parental education, and therefore SES is not described in Table 3. Table 4 shows the changes from baseline to follow-up for all students in Forms 1 through 3. There were no significant differences between Form 3 vs. all students or between schools or sexes.

**Table 3:** Sample characteristics (*n* = 826)

	Mean (SD) or number (%)
	(*n* = 826)^a^
Age	15.65 (1.59)
Sex (male)	460 (56.0)
Ethnicity	
- Maasai	452 (55.9)
- Chagga	85 (10.5)
- Meru	71 (8.8)
- Other	200 (24.7)
Type of toilet at home	
- Pit latrine^b^	596 (73.0)
- No latrine^c^	120 (14.7)
- Bucket latrine	74 (9.1)
- Other	26 (3.2)
Place for hand washing at home (% yes, N)	603 (73.4)
Access to soap/mud/ash (% yes, N)	582 (71.0)

^a^Some variation in denominator due to missing data (no more than 2%)

^b^: type of toilet that collects human feces in a hole in the ground, usually without water

^c^: a bucket is used to collect solid and liquid waste and disposed of elsewhere later (usually an open area, or is buried or used for fertilizer)

**Table 4:** Changes from baseline to follow-up for students in Forms 1 through 3

	Baseline (Mean, SD)^a^	Follow-up (Mean, SD)	Difference	*p* ^c^
Knowledge, Attitudes & Practices				
Knowledge: hygiene ^b^	6.07 (1.59)	6.03 (1.57)	*t*(1,1662) = 0.5	0.614
Perceived severity of worm infection	3.56 (1.36)	3.63 (1.36)	*t*(1,1642) = −0.9	0.330
Perceived importance of washing	2.03 (0.99)	2.15 (1.19)	*t*(1,1657) = −2.3	**0.021**
Frequency of washing	3.91 (0.87)	3.85 (0.91)	*t*(1,1672) = 1.2	0.224
Frequency of unhygienic behaviour	4.50 (0.73)	4.37 (0.81)	*t*(1,1651) = 3.6	**<0.001**
Self-efficacy				
Self-efficacy to wash	4.32 (1.01)	4.27 (1.00)	*t*(1,1647) = 0.9	0.347
Intention to wash	4.37 (0.99)	4.36 (0.98)	*t*(1,1652) = 0.3	0.759
Intention to use toilet	3.78 (1.52)	4.01 (1.42)	*t*(1,1635 = −3.0	**0.003**
Science interest and motivation				
Pros towards science	4.62 (0.58)	4.60 (0.64)	*t*(1,1667) = 0.7	0.504
Cons towards science	2.04 (1.06)	2.20 (1.15)	*t*(1,1651) = −2.9	**0.003**
Communication				
Communication: hygiene	4.11 (0.80)	4.08 (0.84)	*t*(1,1658) = 0.6	0.547
Student Engagement Classroom	4.39 (0.91)	4.40 (0.90)	*t*(1,1667) = 0.2	0.842
Student Engagement Community	3.96 (1.13)	4.15 (1.06)	*t*(1,1661) = −3.6	**<0.001**

^a^ Mean scores from a 5 point Likert scale with 5 indicating higher agreement

^b^ Variable dichotomised and summed for an 8 point scale

^c^ Statistically significant values (<0.05) are in bold

##### Objective 1: knowledge, attitudes and practices related to sanitation and hygiene and increased interest and motivation for science

The quantitative data (Table 4) shows some improvement in terms of changes in knowledge, attitudes and practices. There was a significant increase in the perceived importance of handwashing (*p* <0.001), and a significant decrease in unhygienic behaviours (*p* <0.001). There was also a significant increase in intentions to use the toilet (*p* = 0.003). However, other behaviours and knowledge scales, such as intention to wash and knowledge about hygiene showed no change. In terms of proportional change, the significant results were small. Inspection of the distributions showed that the proportion of students who reported that handwashing before and after eating was ‘very important’, after using the toilet, and after caring for animals, increased marginally from 18.6 to 24%. Similarly, the proportion of students who reported it was ‘very likely’ that they would use a toilet instead of defecating in the open in future increased from 53.6 to 59.4%. For unhygienic behaviours reported in the last 3 months, results showed a marginal decrease from 6.7 to 5.4% in the proportion of students who reported walking barefoot ‘daily’, and a marginal decrease from 5.9 to 5.1% in the proportion of students who reported to defecate outside ‘daily’.

From the qualitative data, students discussed the concepts they had learned including different types of water-borne illnesses, and the different methods for testing water. Students also reported increased knowledge from the experiments they performed in the science fair, including, in particular, discovering that water turbidity could be effectively reduced by filtering it through local cloth that was folded four times.

*And another lesson that we understood well was that on water treatment, for example we learnt how to use filtration instead of using methods, that are not easy to get [not available], even to use filtration, you use a clean piece of cloth, first you filter the water and then you boil until it boils* (SHINE club girls)*.*

Concerning practices within the school environment, one teacher remarked that

*There are some changes in the school policies, for instance our policy of keeping the school environment clean was further strengthened with this project. The relationship also between us and the health care centres because if you take a child to the hospital with abdominal problems they advise the same things that were taught in the project such as ways to prevent diseases* (Teacher 10).

Another teacher commented that students in the SHINE club advocated heavily through school debates for a policy on clean water as a human right, clean latrines and also to end open defecation.

In terms of increased science interest and motivation, the quantitative and qualitative results were contradictory. The quantitative data (Table 4) showed no change in pros towards science, but a statistically significant increase in negative feelings towards science. However, the qualitative data from teachers suggested increased interest in science from students. *“It was very good for the students we were actually glad because it made students gain more interest with the science subjects than before”* (Teacher 2). Students also commented on future aspirations in science with one student remarking that she wanted to be *“the next famous scientist to make an idea, a project just like the Foldscope that can help my community to be healthy”* (SHINE club girls).

##### Objective 2: engage secondary school students and the wider community in the development of innovative sanitation and hygiene strategies

From the quantitative results, there was a significant increase in students’ perceptions that they were engaged in health promotion activities in the community (*p* <0.001) (Table 4). The interviews and focus groups also highlighted student leadership in terms of students being able to replicate lessons they had learned in the classroom to community contexts.

*I found the extra curriculum activities to have been for beneficial to the both the students and the community at large. Students were able to transfer the knowledge they had gotten from the project and share it with the community members* (Teacher 2).

For SHINE club participants, the opportunity to develop leadership skills and engage in peer to peer learning was rewarding and positively impacted the overall school environment. As one student described their experience with SHINE:

*…we were explaining in a group discussion together with our fellow students in the classroom, so I have become more knowledgeable for example that there are some diseases that are caused by animals. Some of us did not know that. So we have learnt that because we shared ideas with our fellow students in the groups. So that has consolidated the relationship and cooperation with our fellow students at school* (SHINE club boys).

Community events, and the sanitation science fair were also viewed as positive experiences which showcased locally relevant solutions, student learning and engaged the community. With regards to the science fair, one teacher noted:

*It was marvelous! The people in the community took part fully together with the teachers and students. A lot of demonstrations and experiments were conducted with valid results which could be seen* (Teacher 7).

### Sustainability of the project

Reflections on the impact of the project raised several important discussions concerning sustainability. One of the SHINE club students commented that the project should be *‘not only for our community in Ngorongoro, but also for other communities in Tanzania. There are communities like this that are not well educated…we should improve it’* (SHINE club boys). Students also reflected on the need for the project to have a wider reach even within their own communities since there are many children who do not attend school. Students expressed widespread appreciation for the relevant focus on frugal strategies to improve health in their communities. For instance, based on instructions provided by Project SHINE, the students constructed tippy taps (simple, low-cost, low-tech handwashing stations) using locally available resources such as wood, rope, buckets and calabashes (gourds used to collect water and milk). Students found that community members and their fellow students were surprised and also *“impressed that we have put them at the toilet, at the laboratory, boys’ toilet, and in different places so that if a person becomes dirty at any point, can go and wash their hands”* (SHINE club girls). This was reflected also in comments by students about the importance of Foldscope as a frugal tool “*The Foldscope is very small that you can put it in your pocket, you can take it anywhere and can look at anything. Different from these big microscopes that are in the laboratory, but these ones you can walk with them, put them in a pocket, cause it is tiny”* (SHINE club girls).

The impact of the project on the future livelihood prospects of participants was highlighted by SHINE club members, with one student reflecting that soap making in particular was an important activity for them to learn.

*This skill, you can also use it even to start your own small industry, you can start making these soap and sell to people, instead of people moving to town where they see that things are cheaper, they can start even moving to villages as they can get this skill* (SHINE club girls).

## Discussion

Project SHINE is an example of an innovative small-scale pilot study which shows promise as a model of engagement and empowerment. Quantitative results were mixed in terms of improving knowledge, practices about sanitation and hygiene, but qualitative data indicate positive changes with respect to these same measures. To the best of our knowledge, this is the first study in the peer-reviewed literature which has presented findings on pastoralist knowledge, attitudes and practices related to an intervention to improve sanitation and hygiene.

### Project implementation

In terms of implementation fidelity and dose, the majority of the activities were carried out as planned, and were widely accepted by teachers, students and community members. However, there were some deviations from the plan, for example with some lessons being delivered on Saturdays instead of during regular class hours. This may have been done when teachers did not believe that the lessons fit into the existing curriculum. However, since both locations are boarding schools where the majority of students remain for the weekends, this is unlikely to have diminished attendance. The train the trainer model was successful as evidenced by the initial training being carried out by five SHINE members and then replicated by 15 teachers and then by 40 students. Participants also noted feeling “ownership” of the knowledge. Similar successes using a train the trainer approach which emphasises community ownership of knowledge have been reported in health promotion interventions elsewhere [30–33]. For example, in Papua New Guinea, a health-promotion intervention was most successful in improving hygiene behaviour when external formally trained health workers worked directly with local village health workers to promote change [30]. In Texas, health promoters working in rural settlements for undocumented migrants succeeded in reducing asthma rates not only by teaching residents to improve ventilation in their homes, but by making these changes in their own homes [32].

### Project outcomes

There were promising findings with respect to the first objective of improving knowledge, attitudes and practices among students related to sanitation and hygiene. These included: reduced frequency of unhygienic behaviour, alongside increased perceived importance of hand-washing; intentions to use the toilet, and engagement in the community. However, other elements such as knowledge about sanitation and hygiene, frequency of washing and intention to use wash hands showed no changes. The lack of changes on these measures, and relatively small changes on other measures that were significant, may be due to several factors. Self-reported questions about sanitation and hygiene are subjective and vulnerable to social desirability bias (the tendency to over-report socially acceptable behaviour), which may have led to an over reporting of hygienic behaviours [12, 24]. This can lead to a ceiling effect with respect to intervention impact, whereby results were already so high at baseline that it was not possible to achieve the difference in scores at follow-up that we had anticipated [24]. It is also possible that gamma change, whereby respondents have re-calibrated the measurement dimension or reconceptualised the behaviour, may have influenced findings [34]. The lack of a control group makes the effect of Project SHINE challenging to objectively assess.

Findings indicate an increased negative attitude towards science education in the quantitative data, which was not supported by the qualitative data. This may have been due to the fact that students who chose to participate in focus groups were also part of the SHINE clubs, and therefore may have had a positive experience with the program. Also, many of the “con” questions in the survey regarding science were negatively worded, which may have led to misinterpretation. For example: “Science I learn about in school is not helpful in my everyday life”.

Contrary to expectations, we did not find any evidence for larger changes between baseline and follow-up in students in Form 3, as these were the students who were supposed to receive the additional school lessons and participated in the sanitation science fair. However, many teachers noted that they had given classes on Saturdays to the whole school, as opposed to just Form 3 students. In addition, the SHINE clubs played a large part in school-based and extracurricular activities, and these clubs included representatives from all Forms who were tasked with sharing their knowledge and skills with their peers. The lack of larger changes in baseline to follow-up in Form 3 compared to the whole school may suggest that the whole school was engaged in the project.

In terms of the second objective of engaging students and community members in developing innovative strategies to address local hygiene and sanitation issues, the Project SHINE model shows substantial promise. Students, teachers and community members enjoyed SHINE activities, and engaged with the project. Some school-based outreach events had over 100 community members participating, including local formal and informal leadership. In a community which is often skeptical of outside intervention, the importance of long-term partnerships with community members cannot be overemphasised [35, 36]. In addition, the One Health approach which seeks to address the interrelatedness of humans, animals and the environment has been shown to be particularly well suited to serve pastoralists, providing an entry point for efforts to improve both human and animal health [37, 38]. The One Health approach in Project SHINE coupled with the underlying positive focus which centered on local knowledge and assets, as opposed to barriers and deficits, appears to have resonated with local community members.

Participatory methods show great potential in terms of mobilizing students and community members to improve sanitation and hygiene. The SHINE clubs in particular, served as focal points at both schools where leadership was fostered and engagement flourished. Similar models using youth health clubs to promote healthy behaviours in Zimbabwe were found to be both cost-effective and acceptable in community settings [39]. We note that teachers selected the SHINE club members, which may have introduced bias as they chose the most motivated students who already demonstrated motivation and leadership capacity. We did not perform cost-effectiveness analyses in this pilot study, but would recommend future studies undertake this in order to assess this component of the intervention.

### Limitations

There were several limitations to this study that need to be noted. The six-month timeframe for the intervention was very short and it was not possible to carry out all activities during this period of time. Elements such as workshops and activities related to social entrepreneurship and the scale up of One Health sanitation science fair projects occurred after the end of the formal intervention. In addition, due to logistical difficulties, the process evaluation was done primarily before the sanitation science fair, which meant that most of the qualitative data did not adequately capture the reflections and learnings from one of the project’s most important events. Other forms of process data that were collected as part of the project such as field notes and digital stories which documented student perceptions and experiences as part of the project were also not incorporated as a data source. In addition, there were scheduling conflicts with other events and school exams that caused unforeseen delays and competition for student and teacher time and resources. Despite the fact that scheduled events had been previously discussed and agreed upon with school headmasters, the fluid nature of academic timetables in Tanzania led to unforeseen scheduling challenges. Teachers also identified lack of time during school hours to provide the lessons. Although all the SHINE lessons had been designed to fit into the existing Tanzanian curriculum so as not to increase teaching burden, teachers may not have as fully bought into this concept. The process evaluation suggested that teachers they felt they were inadequately financially compensated for their involvement in the project. The challenges with time and resources are common in resource-constrained settings [40, 41]. Despite this, there was considerable buy-in from many teachers shown by their willingness to teach additional lessons after school hours, and organise community outreach activities, which may have been possible due to the fact these were boarding schools which may also result in a more controlled environment which influenced the implementation of the intervention. We acknowledge that the schools that participated in this pilot study were relatively well resourced, and other schools that serve pastoralist communities may face additional challenges.

Due to concerns over privacy, the perceived sensitivity of some of the questions and social desirability bias, project partners and school officials recommended that students answered the survey anonymously. This did not allow us to pair the answers specific students gave before and after the intervention. We therefore used unpaired tests in our statistical analysis, which reduced the statistical power to find changes from baseline to follow-up, and limited our ability to account for the correlated nature of the data [42]. A further limitation is the fact that there were only two secondary schools in the intervention area which precluded having a control group and limited our ability to investigate any clustering effect of the schools. However, other studies which focus on health-related behaviours in schools tend to show low-intra-class correlations [43–46].

### Comparison with other approaches

The positive approach of Project SHINE is in contrast to the widely used Community-Led Total Sanitation (CLTS) approach, developed in Bangladesh in the late 1990s. CLTS uses participatory methods and aims to mobilise communities to change their behaviours through eliciting a range of emotional reactions including shame, disgust and dignity to trigger behaviour change and collective action [22]. The most rigorous study to date on the CLTS approach concluded that it may be a promising approach for improving access to sanitation, while others have praised the approach for being community driven and low cost [47]. However, it has also been criticised as being unethical on a number of grounds including the fact that it may contribute to the stigmatization of already marginalised groups [48, 49]. We note that there are other empowering community based approaches that are widely used such as the Participatory Hygiene and Sanitation Transformation (PHAST) model pioneered by the World Health Organization (WHO), UNDP/World Bank Water and Sanitation Program that aims to engage communities to improve water, sanitation and hygiene [50]. In addition, the Children’s Hygiene and Sanitation Training (CHAST) approach was developed in Somalia and uses games and other activities to teach about the link between sanitation, hygiene and health [51].

Project SHINE is explicitly anchored in appreciative inquiry with an aim to identify existing community assets and promote non-stigmatizing activities that empower youth to foster the development of endogenous strategies to improve sanitation and hygiene within the community. The One Health approach to engaging youth and the community in developing sustainable strategies in this pastoralist setting is important to highlight in this regard. The rationale for the emphasis on science education and social entrepreneurship model adopted within SHINE relates to the urgent need to develop innovative strategies to foster resilience and enable and empower communities to develop locally sustainable approaches to improving their education, health and livelihood prospects.

## Conclusion

In this article, we presented the process and outcome evaluation of an innovative pilot project in rural Tanzanian aimed at improving sanitation and hygiene among pastoralists through a science education and social entrepreneurship approach. Although evaluating long term change in terms of the adoption of these sustainable approaches to addressing hygiene and sanitation is beyond the scope of this pilot project, we believe this model shows potential for building capacity and as an engagement and empowerment strategy. Overall, Project SHINE was very well received in schools and the community, and shows promise in terms of changing key sanitation practices, as well as sparking innovation for sustainable change.

## Abbreviations

CHAST: Children’s hygiene and sanitation training
CLTS: Community-led total sanitation
IBM-WASH: Integrated behavioural model for water, sanitation and hygiene framework
MDGs: Millennium development goals
NCA: Ngorongoro conservation area
PHAST: Participatory hygiene and sanitation transformation
SES: Socio-economic status
SHINE: Sanitation and hygiene INnovation in education
WASH: Water, sanitation and hygiene
WHO: World Health Organization
